# Dual-energy X-ray Absorptiometry of Both Hips Helps Appropriate Diagnosis of Low Bone Mineral Density and Osteoporosis

**DOI:** 10.3390/diagnostics7030041

**Published:** 2017-07-09

**Authors:** Pia Afzelius, Mette-Marie Garding, Stig Molsted

**Affiliations:** 1 Department of Diagnostic Imaging, Copenhagen University Hospital, North Zealand Hospital, DK-3400 Hillerød, Denmark; Mette-Marie.Garding@regionh.dk; 2 Department of Clinical Research, Copenhagen University Hospital, North Zealand Hospital, DK-3400 Hillerød, Denmark; Stig.Moelsted@regionh.dk

**Keywords:** osteoporosis, low bone mineral content, bone mineral density, dual-energy X-ray absorptiometry (DXA)

## Abstract

Controversy still remains regarding the use of bilateral hip scanning when bone mineral density (BMD) is measured, and bilateral hip scanning is not mandatory in international guidelines for screening of osteoporosis. BMD of both hips and the lumbar spine was analyzed in 133 consecutive individuals. There were discrepancies between the lowest *T*-scores of both hips. Fourteen of the 133 participants (11%) were diagnosed with a poorer BMD status when the BMD of the hip of the dominant leg was analyzed. The total hip BMD of the dominant hip was lower than in the non-dominant hip, (*p* = 0.035), whereas there were no differences in the femoral neck area of the dominant and the non-dominant leg (*p* = 0.754). When classified by *Z*- or *T*-scores, there was consistency in 60 cases (45%) and inconsistency in 59 cases (44%). In 14 cases (11%), *T*- or *Z*-scores were the same, and it did not matter whether the non-dominant hip or the dominant hip had been chosen. A diagnostic discordance of 11% between the left and the right hip was observed when the lumbar spine was evaluated. The lowest *Z*- and *T*-scores of the hips were, in 44% of the cases, found in the hip of the assumed dominant leg. BMD measurements of both hips are recommended as clinical practice.

## 1. Introduction

Osteoporosis (OP) is defined as a systemic disease of the bones characterized by low bone mass and micro-changes in bone formation leading to increased fragility and an increased risk of fracture [1]. Bone mass is the most important predictor of bone strength. Thus, approximately 80% of bone strength is directly related to bone mineral density (BMD) [2,3,4], while the remaining part can be explained by the elasticity of the bone tissue, which depends on the bone matrix and the bone tissue three-dimensional architecture, including bone geometry [5]. OP is a common clinical disorder and a major health care problem due to the considerable annual number of fractures that is related to it. The National Institute of Health reports that 10 million individuals in the U.S. already have osteoporosis and 34 million more have low bone mass, placing them at increased risk of this disease. One of every two women and one in four men over 50 years of age will have an osteoporosis-related fracture in their lifetime. Osteoporosis is estimated to be responsible for 300,000 hip fractures annually. More than 2 million American men suffer from osteoporosis, and millions more are at risk. Approximately 25% of hip fractures occur in men. The OP prevalence in Scandinavian countries is higher than in other European countries. As per the World Health Organization (WHO) definition of OP [6], 500,000–600,000 Danes over the age of 50 years have OP, corresponding to 10% of the total population.

Dual-energy X-ray absorptiometry (DXA) is together with X-ray of the spine the gold standard for determining BMD due to its precision. In addition, DXA implies a low radiation dose which makes it useful in clinical practice. BMD is usually measured at the lumbar spine (L1–4) and in the total hip and the femoral neck to grade the severity of low bone density (LBD) and OP by the lowest *T*-score. The *T*-score is defined as the number of standard deviations from the mean value for healthy 30-year-old Caucasian women, and the *Z*-score is the number of standard deviations from the mean of the patient’s age, sex, and ethnicity. A *T*-score of −1.0 or higher is categorized as normal BMD. A *T*-score between −1.0 and −2.5 means LBD, and a *T*-score of −2.5 or lower is considered diagnostic of OP. A *Z*-score of −2 or lower may suggest an abnormal bone loss due to factors apart from aging. 

Unilateral measurements of proximal femoral BMD are typically performed to minimize the time, medical costs, and radiation exposure is associated with radiography. Moreover, the measurement of one hip is based on the assumption that there are no significant BMD differences between the two hips. Whilst various studies have suggested that there are no significant differences in BMD between the right and left hip, and that leg dominance does not have an influence on the BMD of any of the hip regions, other studies have found differences. As per the International Society for Clinical Densitometry Guidelines 2015, scanning of both hips has not yet been established [7,8]. It is known that the BMD in the dominant forearm exceeds that found in the non-dominant forearm. Thus, it is therefore agreed that examining the non-dominant forearm ensures measurement of the lowest BMD value of a subject. Regarding the hips, it remains unclear whether there also is a systematic difference here [7,8]. In Denmark, it is common to scan the hip of the same side as the non-dominant arm. We thus wanted to determine the diagnostic agreement and the degree of misclassification when using data of the left and the right hip.

## 2. Material and Methods

### 2.1. Participants

BMD in both hips and the lumbar spine was measured with DXA in 133 consecutive women (*n* = 115) and men (*n* = 18) between 30 and 90 years of age. 

### 2.2. Ethics

The invited participants received written and oral information about the study and gave their informed consent to participate in the study, which was in accordance with rules of the local ethics committee and and The Danish Data Protection Agency (j.nr.:2012-58-0004, 14 June 2016).

### 2.3. DXA

Both hips and the lumbar spine were examined using a Hologic Discovery Delphi SL, QDR^®^ Series (Hologic, Inc., Bedford, MA, USA) with dual-energy X-ray. Adult whole-body software (QDR v. 12.1, http://www.hologic.com/support/discovery-dxa-system; Hologic, Inc., Bedford, MA, USA) was used for data acquisition and analysis. The Third National Health and Nutrition Examination Survey, NHANES III, data were used at the reference standard for *T*- and *Z*-scores in the femoral neck and the total hip areas. The manufacturer’s reference data were used for the lumbar spine analysis. Bone mineral densities were expressed as grams per square centimeter (g/cm^2^). The bone status was determined by the lowest *T*-score. The participants’ BMD was classified as normal, LBD, osteoporotic bone, or severely osteoporotic bone according to WHO criteria [6]. Both DXA acquisition and evaluation of the results were performed according to the International Society for Clinical Densitometry (ISCD) 2015 Guidelines. Additionally, age, height, weight, body mass index (BMI), time of menopause, and dominant hip and arm were recorded.

### 2.4. Statistics

Statistical analyses were performed using the Statistical Package for the Social Sciences (SPSS) v.22 software (IBM Corp., Armonk, NY, USA). The distributions of the data and residuals were tested using *Q*–*Q* plots and histograms. Student’s paired *t*-test was used to compare BMD data between the individual participants. Bivariate correlations were tested using Pearson’s correlation test. Data are presented as the mean ± standard deviation (SD), counts and percentages, or correlation coefficients. All tests were two-tailed and *p* ≤ 0.05 was considered significant.

## 3. Results

Characteristics of the 133 participants are presented in Table 1. The participants were stratified into four groups: post-menopausal females; non-menopausal females; males ≥50 years of age; and males <50 years of age.

There were no differences between right and left BMD overall in participants and in the subgroups of participants. In all participants, the spine BMD exceeded the BMD of the left and right hip neck and the total hip (*p* ≤ 0.001).

**Figure 1:**
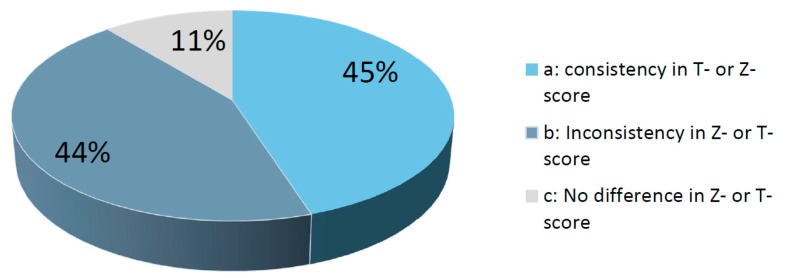
Percentage of cases when: (**a**) the non-dominant leg had the lowest *Z*- or *T*-score; (**b**) the *Z*- or *T*-score was lowest in the dominant leg; and (**c**) it did not matter which leg was chosen since the *T*- or *Z*-score was the same in both legs.

**Figure 2:**
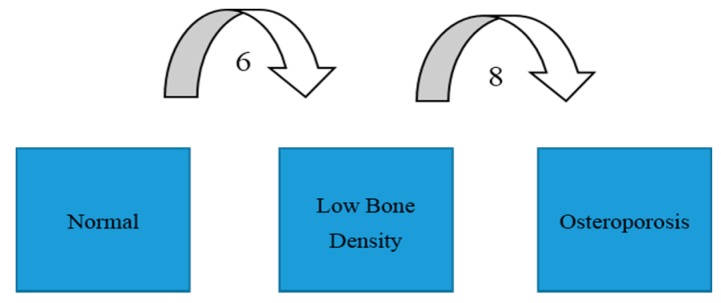
For 14 of the patients (11%), scanning of both hips turned out to have diagnostic significance.

The dominant hand did not always match the highest or *Z*-score at the femoral neck on the same side (Figure 1). It was correct in 60 cases (45%), whereas it was lower in 59 cases (44%). In 14 cases (11%), it did not matter whether the hip of the dominant or non-dominant leg was chosen as *Z*- and *T*-score was the same in both legs. In all participants, the total hip BMD was lower in the dominant leg than in the non-dominant leg, with values of 0.825 ± 0.133 g/cm^2^ vs. 0.832 ± 0.133 g/cm^2^, respectively (*p* = 0.035). There was no difference between the femoral neck BMD of the dominant and the non-dominant legs, with values of 0.706 ± 0.113 g/cm^2^ vs. 0.707 ± 0.115 g/cm^2^, respectively (*p* = 0.754). The self-reported non-dominant leg did not make the picture clearer because in seven cases out of 133 the non-dominant hip had higher *T*- or *Z*-scores than the dominant hip, and in eight cases using self-reported non-dominant hip, the *T*- or *Z*-scores were lower than in the dominant hip. In Table 2, the results of the standard diagnoses with BMD analyses of the non-dominant hip are compared with the results of the diagnoses based on BMD analyses with BMD of the other hip included. Six participants (~5%) of those with a normal BMD in the non-dominant hip had low BMD in the dominant hip, and eight participants (6%) of those with LBD in the non-dominant hip had OP in the dominant hip (Figure 2). Thus, 14 (12 postmenopausal females and two males ≥50 years) of the 133 participants (~11%) were diagnosed with a poorer BMD status when the data of both hips were analyzed. In five participants, the BMD category status was impaired when the result of the additional analyzed hip was compared to that of the standard tested hip and the lumbar spine.

**Figure 3:**
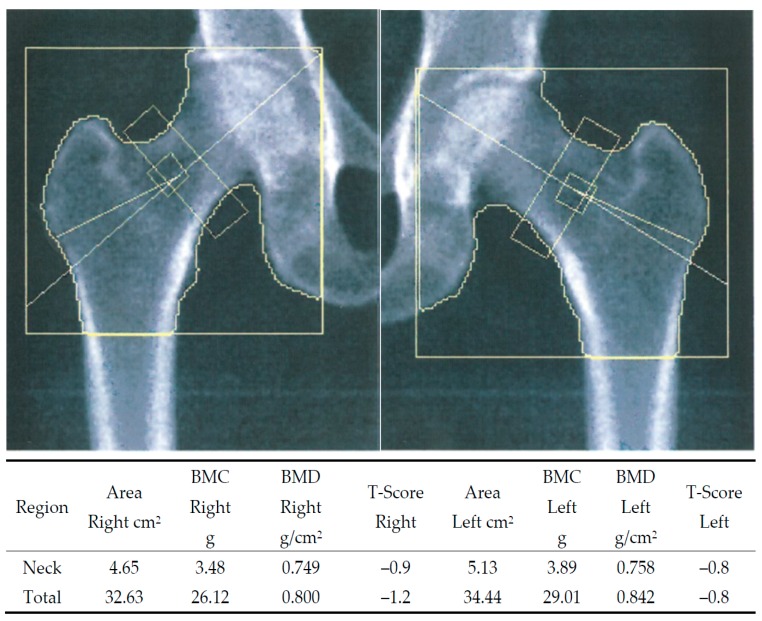
Bilateral hip DXA scan image from a 59-year-old post-menopausal woman. The dominant arm did not match, but dominant leg did. The *T*-score for the lumbar spine was normal. If the patient had only had her left hip examined in accordance with the dominant arm, the conclusion would have been normal bone mineral density (BMD). Having both hips examined instead led to the conclusion of low bone density (LBD).

In one case, a patient had normal BMD in the lumbar vertebrae. If the patient had only had her left hip scanned, she would had been evaluated as having normal BMD, but on scanning the dominant hip as well a diagnosis was LBD was made (Figure 3).

All BMD data of the hips and the spine were negatively associated with participant age and positively associated with height, weight, and body mass index (BMI; Table 3).

## 4. Discussion

The presence of OP predicts fracture risk. Fragility fractures are associated with marked morbidity as well as mortality. Thus, OP has marked therapeutic and economic implications. It is therefore important to categorize the patients correctly in order to provide an appropriate preventive and medical therapy to avoid the occurrence of a low-energy fracture. The gold standard technique for estimation of BMD is the DXA technique due to its reproducibility, large normative data, non-invasive nature, short procedure time, and minimal radiation exposure [9,10]. In some cases, it may sometimes be problematic to use two-dimensional imaging of a three-dimensional structure as the legs may not be anatomically identical or it may be impossible to position the hips similarly, but making sure that the areas of interest are of the same size and that positioning of the individual hips of the patient is similar at follow-ups on the same scanner will ensure that the patient serves as his/her own baseline. For a clinical perspective, we used the lowest *T*- or *Z*-score to determine bone health according to the WHO classifications. Measurement of four vertebrae, if possible, improves diagnosis compared to measurement of three vertebrae, as does a measurement of both hips compared to only one hip. The value of examining both hips has not been established. A hip fracture causes substantially greater morbidity, mortality, and economic costs than fractures of the lumbar spine and the wrist [11]. In the present study, eight participants (20%) of those with a normal BMD in the non-dominant hip had low BMD in the dominant hip, and eight participants (10%) of those with LBD in the non-dominant hip had OP in the dominant hip. Scanning both hips thus changed the treatment strategy towards prevention of fragile fractures. The reasons for discordance between the hips may be multiple: genetics, dominance, immobilization, training, working procedures, osteoarthrosis/-arthritis, illnesses, etc. The study design of consecutive patient inclusion will favor post-menopausal women. The other three groups were too small for definite comparisons between groups’ age and sex. A comparison of side differences, however, seems rational since the individuals were their own controls. 

In our study, we furthermore found that the BMD of the total hip as well as of the femoral neck was lower in the assumed dominant leg than in the non-dominant leg. Moreover, we found that it was not enough to use the self-reported non-dominant hip. This supports full examination of both the hips and the femoral necks of both hips, especially when the lumbar spine is normal or it is not interpretable (e.g., in osteoarthritis).

## 5. Conclusions

There is no significant difference between the dominant and the non-dominant femoral neck, but in more than 10% of the scanned patients, it was found to have a critical diagnostic importance. The *Z*- or *T*-score in the assumed dominant leg was often (in 44% of cases) lower than in the non-dominant leg. It would be more correct to scan both the right and the left femoral neck and total hip at the first DXA scan, especially if the spine cannot be evaluated. Analyzing the self-reported non-dominant hip did not improve the results. BMD measurements of both hips are therefore recommended, ensuring the diagnosis of LBD and OP in non-osteoporotic individuals when the lumbar spine *T*-score is above −2.5 or is not interpretable. BMD measurements of both hips are suggested for screening and diagnostic purposes in clinical practice, particularly in post-menopausal women in order to institute correct and sufficient prophylactic and medical treatment.

## Figures and Tables

**Table 1:** Patient characteristics and results of the dual X-ray absorptiometry (DXA) scan. BMD, bone mineral density; SD, standard deviation.

Variables	All Participants (*n* = 133)	Post-Menopausal Females (*n* = 101)	Non-Menopausal Females (*n* = 14)	Males <50 Years (*n* = 3)	Males >50 Years (*n* = 15)
Age (years)	63 ± 11	66 ± 8	45 ± 6	41 ± 10	68 ± 8
Gender, female (%)	86.5	100	100	0	0
Height (cm)	165 ± 8	163 ± 6	165 ± 7	178 ± 3	178 ± 9
Weight (kg)	70 ± 14	67 ± 13	69 ± 18	93 ± 14	80 ± 12
Body mass index (kg/m^2^)	25.5 ± 4.7	25.4 ± 4.7	25.5 ± 6.1	29.2 ± 3.7	25.3 ± 3.6
Right femoral neck (g/cm^2^)	0.71 ± 0.11	0.69 ± 0.10	0.84 ± 0.12	0.81 ± 0.15	0.69 ± 0.09
Left femoral neck (g/cm^2^)	0.71 ± 0.12	0.69 ± 0.11	0.82 ± 0.12	0.81 ± 0.13	0.70 ± 0.10
Right hip total (g/cm^2^)	0.83 ± 0.13	0.80 ± 0.12	0.94 ± 0.15	0.95 ± 0.16	0.87 ± 0.11
Left hip total (g/cm^2^)	0.83 ± 0.13	0.81 ± 0.12	0.94 ± 0.15	0.96 ± 0.13	0.88 ± 0.12
Dominant femoral neck (g/cm^2^)	0.71 ± 0.11	0.69 ± 0.10	0.83 ± 0.12	0.80 ± 0.14	0.69 ± 0.09
Non-dominant femoral neck (g/cm^2^)	0.71 ± 0.11	0.69 ± 0.11	0.82 ± 0.12	0.81 ± 0.13	0.70 ± 0.10
Dominant hip total (g/cm^2^)	0.83 ± 0.13	0.80 ± 0.12	0.94 ± 0.15	0.94 ± 0.15	0.87 ± 0.11
Non-dominant hip total (g/cm^2^)	0.83 ± 0.13	0.81 ± 0.12	0.94 ± 0.15	0.97 ± 0.13	0.88 ± 0.12
Right femoral neck ( *T*-score)		−1.46 ± 0.93			−1.73 ± 0.67
Left femoral neck ( *T*-score)		−1.42 ± 0.99			−1.71 ± 0.71
Right hip total ( *T*-score)		−1.19 ± 1.00			−1.07 ± 0.75
Left hip total ( *T*-score)		−1.12 ± 0.99			−1.03 ± 0.78
Right femoral neck ( *Z*-score)			0.44 ± 1.11	−0.40 ± 1.35	
Left femoral neck ( *Z*-score)			0.26 ± 1.10	−0.40 ± 1.18	
Right hip total ( *Z*-score)			0.35 ± 1.32	−0.40 ± 1.14	
Left hip total ( *Z*-score)			0.34 ± 1.24	−0.30 ± 0.95	
Lumbar spine (g/cm^2^)	0.92 ± 0.16	0.90 ± 0.15	1.08 ± 0.17	0.99 ± 0.20	0.94 ± 0.14
Lumbar spine ( *T*-score)		−1.57 ± 1.27			−1.43 ± 1.36
Lumbar spine ( *Z*-score)			0.56 ± 1.70	−0.93 ± 2.00	
BMD non-dominant hip					
Normal (%( *n*))	28.6 (38)	21.8 (22)	78.6 (11)	66.7 (2)	20.0 (3)
Low bone density (%( *n*))	60.2 (80)	64.4 (65)	21.4 (3)	33.3 (1)	73.3 (11)
Osteoporosis (%( *n*))	11.3 (15)	13.9 (14)	0 (0)	0 (0)	6.7 (1)
Menopause duration (years)		16 ± 9			

Data are presented as mean ± SD, number and percentage.

**Table 2:** Bone mineral density status in the non-dominant hip vs. the dominant hip.

BMD Non-dominant Hip	BMD Dominant Hip		
	Normal	LBD	Osteoporosis
Normal (*n* (%))	32 (24.1)	6 (4.5)	0 (0.0)
LBD (*n* (%))	10 (7.5)	62 (46.6)	8 (6.0)
Osteoporosis (*n* (%))	0 (0.0)	5 (3.8)	10 (7.5)

Data distribution between the categorizations of the two hip analyses were significantly different, *p* < 0.001. BMD, bone mineral density; LBD, low bone density.

**Table 3:** Correlations between participant age, menopause duration, height, weight, body mass index (BMI), and BMD.

Variable	Right Hip Neck (g/cm^2^)	Left Hip Neck (g/cm^2^)	Right Hip Total (g/cm^2^)	Left Hip Total (g/cm^2^)	Lumbar Spine (g/cm^2^)
Age (years)	*r* =−0.402 *p* < 0.001	*r* = −0.422 *p* < 0.001	*r* = −0.354 *p* < 0.001	*r* = −0.375 *p* < 0.001	*r* = −0.339 *p* < 0.001
Menopause duration (years)	*r* = −0.314 *p* = 0.004	*r* = −0.394 *p* = 0.001	*r* = −0.358 *p* = 0.001	*r* = −0.433 *p* < 0.001	*r* = −0.447 *p* < 0.001
Height (cm)	*r* = 0.209 *p* = 0.016	*r* = 0.249 *p* = 0.004	*r* = 0.289 *p* = 0.001	*r* = 0.295 *p* = 0.001	*r* = 0.275 *p* = 0.001
Weight (kg)	*r* = 0.387 *p* < 0.001	*r* = 0.435 *p* < 0.001	*r* = 0.493 *p* < 0.001	*r* = 0.537 *p* < 0.001	*r* = 0.309 *p* < 0.001
Body mass index (kg/m^2^)	*r* = 0.338 *p* < 0.001	*r* = 0.349 *p* < 0.001	*r* = 0.428 *p* < 0.001	*r* = 0.462 *p* < 0.001	*r* = 0.217 *p* = 0.012
Right hip neck (g/cm^2^)		*r* = 0.923 *p* < 0.001	*r* = 0.883 *p* < 0.001	*r* = 0.847 *p* < 0.001	*r* = 0.683 *p* < 0.001
Left hip neck (g/cm^2^)			*r* = 0.845 *p* < 0.001	*r* = 0.861 *p* < 0.001	*r* = 0.656 *p* < 0.001
Right hip total (g/cm^2^)				*r* = 0.956 *p* < 0.001	*r* = 0.670 *p* < 0.001
Left hip total (g/cm^2^)					*r* = 0.681 *p* < 0.001

Data are presented as the correlation coefficient *r*.
